# Prolotherapy as a Regenerative Treatment in the Management of Chronic Low Back Pain: A Systematic Review

**DOI:** 10.3390/medicina61091588

**Published:** 2025-09-02

**Authors:** Stelian-Ilie Mociu, Andreea-Dalila Nedelcu, Andreea-Alexandra Lupu, Andreea-Bianca Uzun, Dan-Marcel Iliescu, Elena-Valentina Ionescu, Madalina-Gabriela Iliescu

**Affiliations:** 1 Faculty of Medicine, Doctoral School, “Ovidius” University of Constanta, 900470 Constanta, Romania; mociustelian@gmail.com (S.-I.M.); dalila.nedelcu@365.univ-ovidius.ro (A.-D.N.); bianca.uzun@365.univ-ovidius.ro (A.-B.U.); dan.iliescu@univ-ovidius.ro (D.-M.I.); elena.ionescu@365.univ-ovidius.ro (E.-V.I.); 2 Department of Physical Medicine and Rehabilitation, Balneal and Rehabilitation Sanatorium of Techirghiol, Techirghiol, 906100 Constanta, Romania

**Keywords:** chronic low back pain, prolotherapy, medical rehabilitation, quality of life

## Abstract

*Background*: Chronic low back pain markedly impairs quality of life and imposes a significant economic burden on public health. The complex pathophysiology of chronic low back pain arises from the complex anatomical configuration of the lumbar region, which includes a diverse array of structures. Consequently, etiologies may involve intervertebral disc degeneration, facet joint osteoarthritis, spinal stenosis, spondylosis, and spondylolisthesis. Therapeutic interventions for chronic low back pain are equally varied, ranging from pharmacological treatments to physiotherapy, kinetotherapy, balneotherapy, and image-guided local injectable procedures such as prolotherapy. Prolotherapy is a regenerative injection technique designed to stimulate the body’s healing processes by applying a regenerative treatment (typically dextrose), which aims to modulate neurogenic inflammation and diminish nociceptive signaling. *Methods*: A systematic review of the literature was performed in alignment with the PRISMA guidelines (Preferred Reporting Items for Systematic Reviews and Meta-Analyses). Studies published within the last ten years evaluating the effects of prolotherapy on pain reduction in individuals with chronic low back pain were included, following a search across six databases. Results: The review revealed several studies evaluating the influence of prolotherapy on pain in chronic low back pain patients. Findings were heterogeneous, with some studies indicating significant pain reduction and others showing minimal or no improvement. *Conclusions*: The current evidence regarding the efficacy of prolotherapy for pain relief in chronic low back pain remains inconclusive, highlighting the necessity for further in-depth research. Continued and updated investigations into prolotherapy’s role are imperative for enhancing the quality of life of affected patients.

## 1. Introduction

### 1.1. Definition of Chronic Low Back Pain (CLBP)

Chronic low back pain (CLBP) is defined as pain localized below the costal margin and above the inferior gluteal folds that persists for more than 12 weeks, with or without leg pain or neurological symptoms [1]. This condition is considered “chronic” when the pain continues beyond the expected period of healing, typically three months, and may fluctuate in intensity over time [2]. CLBP arises from a variety of etiological mechanisms, which can be broadly grouped according to the presence or absence of identifiable structural pathology [1]. The most prevalent form is non-specific low back pain, representing approximately 85–90% of all cases in the absence of a detectable anatomical cause after thorough evaluation [1,3].

CLBP is widely recognized as a prevalent and debilitating condition with a multifactorial etiology. In addition to the categorization based on structural pathology, the condition is understood to arise from a complex interaction of biomechanical factors, such as degenerative changes, muscle imbalances, cumulative microtrauma, and psychosocial elements, including anxiety, depression, and maladaptive coping strategies [3,4,5].

### 1.2. Pathophysiological Mechanisms and Etiology of CLBP

Intervertebral disc degeneration is frequently implicated in discogenic pain, as degenerative disc disease leads to loss of disc height, annular fissures, reduced shock absorption, and internal disc disruption that may provoke nociceptive signaling from the annulus fibrosus [6,7]. Facet joint arthropathy, lumbar disc herniation, spinal stenosis. sacroiliac (SI) joint dysfunction, spondylolisthesis, vertebral fractures, infectious processes, neoplasms, and inflammatory conditions, such as axial arthritis, represent etiological factors implicated in CLBP [8,9,10,11,12,13,14]. The majority of cases are predominantly considered to be mechanically mediated, with pain exacerbated by movements or sustained postures and modulated through both central and peripheral sensitization processes [15,16].

### 1.3. Epidemiology and Public Health Impact of CLBP

According to the Global Burden of Disease (GBD) 2021 data, over 619 million people were affected globally, with estimates suggesting that this number will increase to 843 million by 2050 [17]. The lifetime prevalence of CLBP is estimated at 60–80%, while point prevalence ranges from 12% to 33% across different populations [18]. Risk increases with age, reaching a peak in individuals aged 50–60 years, and women tend to report a higher incidence of CLBP than men [19]. Moreover, CLBP imposes a substantial economic burden due to direct medical costs and lost productivity [20,21]. Beyond its financial implications, CLBP significantly reduces quality of life by impairing mobility and contributing to depression, anxiety, and social isolation [22].

### 1.4. Risk Factors for CLBP

CLBP results from a complex interaction of biomechanical, psychosocial, occupational, and lifestyle-related factors [23]. Its incidence increases with age due to progressive degenerative spinal changes and diminished muscular support [24]. Women tend to exhibit a slightly higher prevalence of CLBP, particularly after menopause, potentially owing to hormonal influences and differences in pain processing pathways [25,26]. Occupational exposures such as repetitive bending, heavy lifting, prolonged static postures, or exposure to vibration are known as risk factors for developing CLBP [27]. Additional contributors include excess body weight, smoking, sedentary behavior poor postural control, psychological factors such as depression, anxiety, catastrophizing, job dissatisfaction, and low socioeconomic status [24,28,29,30,31,32,33,34]. Early identification of these risk factors is essential for implementing targeted prevention strategies and designing personalized rehabilitation programs [22,32].

### 1.5. Prolotherapy

Prolotherapy (dextrose prolotherapy), an abbreviated term for proliferation therapy, is an injection-based regenerative treatment designed to stimulate the body’s natural healing processes by inducing a controlled inflammatory cascade [35]. The concept originated in the 1930s when Dr. Earl Gedney, an osteopathic physician, proposed that ligament laxity played a significant role in musculoskeletal pain, and this technique was later refined and widely disseminated in the 1950s by Dr. George Hackett, whose extensive clinical experiences laid the foundation for modern prolotherapy [35,36]. Initially, treatment protocols employed mixtures such as phenol-glycerin-glucose; but modern protocols primarily use hypertonic dextrose (typically 12.5–25%) for its safety and efficacy [37]. Unlike corticosteroid injections, which suppress inflammation, prolotherapy promotes tissue regeneration by activating fibroblasts and enhancing collagen deposition, and leads to tissue remodeling [35,38,39].

### 1.6. Mechanisms of Prolotherapy

The precise biological mechanism of prolotherapy (dextrose prolotherapy) remains incompletely understood. It is theorized that injection with hypertonic dextrose induces osmotic stress and cellular dehydration where it is injected, triggering a cascade involving granulocytes and macrophages and subsequent release of growth factors [40]. The proliferative phase enhances the production of collagen types I and III, while neovascularization restores tissue perfusion [37]. The remodeling phase realigns collagen fibers, resulting in improved tissue integrity and function [41,42]. Moreover, prolotherapy has been shown to modulate nociceptive signaling and neurogenic inflammation, contributing to pain relief [43,44].

### 1.7. Therapeutic Uses of Prolotherapy (Dextrose Prolotherapy)

Prolotherapy (dextrose prolotherapy) has demonstrated efficacy in a broad range of chronic musculoskeletal conditions in which dysfunction of ligaments or tendons plays a central role in pain and instability [45]. Frequent clinical indications include knee osteoarthritis lateral epicondylitis, Achilles tendinopathy plantar fasciitis, and temporomandibular joint dysfunction [43,45,46,47,48,49,50,51]. In many cases, prolotherapy (dextrose prolotherapy) is administered following the failure of conservative treatments, including physical therapy, non-steroidal anti-inflammatory drugs (NSAIDs), or corticosteroid injections [37].

### 1.8. Prolotherapy (Dextrose Prolotherapy) in CLBP

In the context of CLBP, prolotherapy (dextrose prolotherapy) is utilized to target the ligamentous and connective tissue structures that contribute to segmental instability, pain sensitization, and degenerative overload [52,53]. Common target structures include the supraspinous and interspinous ligaments, iliolumbar ligaments, facet joint capsules, sacroiliac joint ligaments, thoracolumbar fascia, and the paraspinal tendinous insertions of muscles such as the multifidus and erector spinae [53]. Although some clinicians perform injections based exclusively on anatomical landmarks, image guidance (using ultrasound) is strongly recommended to enhance accuracy and safety [54]. Standard protocols typically involve the administration of hypertonic dextrose (12.5–25%), often combined with a local anesthetic such as lidocaine, sessions repeated every 2 to 4 weeks, and a total of three to six treatments adapted to clinical response [54]. Indications for prolotherapy (dextrose prolotherapy) in CLBP include non-specific CLBP, sacroiliac joint dysfunction, facet-mediated pain, degenerative disc disease with associated ligamentous laxity, and post-traumatic lumbar hypermobility [55].

### 1.9. Aim of the Review

Considering the existing information in the literature, this systematic review aims to explore the effects of prolotherapy on patients with CLBP by evaluating both symptom relief (pain reduction) and improvement in lumbar mobility. It aims to answer the following questions: “What is the effectiveness of prolotherapy in patients with CLBP, how effective is it in the long term, and how does it impact their quality of life?”. Following the PICO model, this systematic review focuses on patients with CLBP (P), the use of prolotherapy as a treatment intervention (I), compared to placebo or conventional therapies (C), with outcomes measured in terms of pain reduction, improved lumbar mobility, long-term effectiveness, and enhanced quality of life (O).

## 2. Materials and Methods

This systematic review was carried out following the internationally recognized PRISMA guidelines to ensure standardized reporting [56]. The protocol was also prospectively registered in PROSPERO (International Prospective Register of Systematic Reviews) under the registration number CRD420251101779.

The searches were conducted in the following databases to retrieve studies relevant to the investigation: PubMed, ScienceDirect, Scopus, Springer Nature, Web of Science, and Cochrane. In the literature, prolotherapy is described using various terms, including hypertonic glucose, dextrose prolotherapy, and D-glucose prolotherapy. All of these variants were included in the search strategy, which incorporated the following keywords: “prolotherapy” OR “hypertonic glucose” OR “dextrose prolotherapy” OR “D-glucose prolotherapy” AND “low back pain”.

We used the term “low back pain” because many key studies do not include the word “chronic” in their titles or indexing. LBP is among the most prevalent musculoskeletal disorders worldwide, and the reporting symptoms are beyond three months, thereby meeting most definitions of chronicity [57,58]. Yet, how “chronic low back pain” is defined varies: some guidelines set the threshold at 12 weeks, others at 6 months, and some simply group all persistent symptoms under LBP without a time qualifier [4]. As a result, numerous publications addressing long-term or recurrent LBP omit the term “chronic” although they refer to chronic pain [4]. Moreover, the World Health Organization defines LBP broadly to include acute, subacute, and chronic phases of pain, meaning that persistent symptoms are often discussed under the umbrella term of “low back pain” [58]. Therefore, we consider that restricting the search to “chronic low back pain” would risk missing important studies, whereas using the broader term “low back pain” ensures all relevant chronic pain articles are captured.

Our research was limited to studies published between January 2015 and March 2025, written in English. We excluded review articles, editorials, or studies involving non-human subjects. Additionally, we did not consider articles written in languages other than English, publications older than 10 years, book chapters, full books, or conference abstracts.

### 2.1. Inclusion Criteria

− Original studies (randomized controlled trials, cohort studies, experimental studies, or observational designs) evaluating the effects of prolotherapy (dextrose prolotherapy) in patients with CLBP;
− Studies assessing outcomes related to pain intensity, lumbar mobility, or quality of life following prolotherapy (dextrose prolotherapy);
− Studies involving adult or elderly participants of any age, no age restriction applied;
− Studies including CLBP of mechanical cause, degenerative (facet joint degeneration, lumbar spinal stenosis), disc herniation—regardless of surgical status, failed back surgery syndrome, sacroiliac joint dysfunction, non-specific causes;
− Studies published in English;
− Studies published within the last 10 years.

### 2.2. Exclusion Criteria

− Systematic reviews, meta-analyses, case reports, editorials, letters to the editor, conference abstracts, and book chapters;
− Studies involving patients with inflammatory, infectious, or neoplastic or post-traumatic causes of low back pain;
− Studies focused on pediatric populations;
− Animal studies or preclinical research without clinical application;
− Studies published in languages other than English;
− Studies published before 2015.

The selection process was conducted by two independent reviewers who screened each record to determine eligibility based on the predefined inclusion and exclusion criteria. Any discrepancies were resolved through discussion or consultation with a third reviewer.

Data extraction was carried out independently by three reviewers using a standardized form. Discrepancies among reviewers were addressed through discussion until consensus was reached.

The risk of bias in the included studies was independently assessed by two reviewers using two validated tools: the Cochrane Risk of Bias 2 (RoB 2) tool for randomized controlled trials and the PEDro Scale, with any discrepancies between assessments resolved through discussion or, when necessary, with input from a third reviewer.

## 3. Results

**Figure 1:**
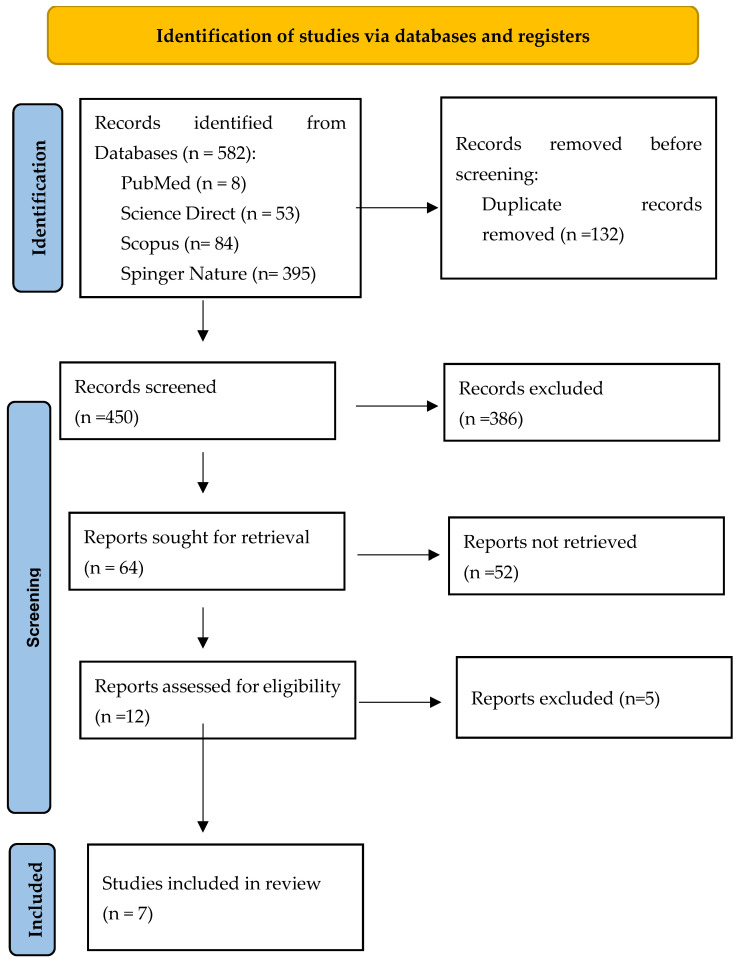
PRISMA flow diagram.

A total of 582 records were initially identified, as shown in Table 1. These records were imported into Zotero (version 7.0.15) reference management software, where 132 duplicates were detected and subsequently removed. The remaining articles underwent a rigorous eligibility assessment. Following the screening of titles and abstracts, 386 articles were excluded for not meeting the preliminary inclusion criteria. Of the 64 articles selected for evaluation, 52 were excluded due to lack of relevance to the specific objectives of our review. Among the remaining 12 articles, an additional five were excluded as they were case–control studies, which did not align with our predefined study design criteria. After evaluating eligibility, seven studies were selected for inclusion in this systematic review (Figure 1).

The included studies’ descriptions are synthesized in Table 2, which outlines key information. These studies were retained because the topic remains under debate in the literature and was considered to require a systematic review to identify the most appropriate approach, despite significant gaps in the current research.

### 3.1. Short Description of the Included Studies

The study conducted by Liza Maniquis-Smigel et al. (2017) aimed to evaluate the short-term analgesic effect of a single 10 mL caudal epidural injection of 5% dextrose (D5W) compared with 0.9% saline in patients suffering from moderate to severe CLBP with radiation into the buttocks or legs [59]. Thirty-seven participants were randomized to receive either D5W (19 patients) or saline (16 patients), using a caudal epidural injection technique. Pain intensity was measured using a 0–10 Numeric Rating Scale (NRS) at multiple intervals: baseline, 15 min, 2 h, 4 h, 48 h, and 2 weeks post-injection [59].

The primary outcome was measuring pain with the NRS, and secondary outcomes included the percentage of patients achieving >50% pain reduction at 4 h post-injection. Also, the results showed that the D5W group experienced significantly greater reductions in pain at every time point through 48 h. Additionally, 84% of D5W group patients versus only 19% of saline group patients reported at least 50% pain reduction at 4 h. Pain scores at 2 weeks were not statistically different between the two groups. This study demonstrates a statistically and clinically significant short-term analgesic effect following a single dextrose injection [59].

The study conducted by Özlem Köroğlu et al. (2019) investigated the clinical efficacy of 5% dextrose prolotherapy in patients presenting with chronic radicular low back pain secondary to lumbar disc herniation [60]. The study enrolled 40 patients who had been symptomatic for more than three months and the diagnosis was imaging-confirmed for disc herniation, without prior spinal surgery or infiltrative interventions. Patients were divided into two groups, equal in number: one received prolotherapy alone, while the other one received prolotherapy in conjunction with a standardized physical therapy program, which consisted of TENS therapy, infrared rays, and stretching exercises [60]. Both groups underwent three sessions of prolotherapy (dextrose prolotherapy) at four-week intervals, with injections targeted to the iliolumbar and transverse ligament insertions as well as the lumbar facet joints. Pain medication was limited to paracetamol, and NSAIDs were discontinued to support the intended inflammatory-regenerative response. The follow-up was performed at 3, 12, and 52 weeks after the first injection [60]. Clinical outcomes were assessed using the VAS, the ODI, and the SF-36 for quality of life questionnaire at baseline, three weeks, twelve weeks, and one year post-treatment [60]. Both groups demonstrated statistically significant improvements in all measured outcomes at each follow-up point compared to baseline, with the effects sustained for up to one year [60]. However, no statistically significant differences were observed between the two groups throughout the follow-up period, suggesting that the addition of physical therapy did not enhance the effects of prolotherapy in this specific protocol [60]. The study lacks a control group and has a small number of patients; the physical therapy has very few therapies associated, but its outcomes align with the prior literature that supports the analgesic and potentially neurogenic modulation effects of 5% dextrose [60].

In a study published in 2021, Yildirim compared the clinical effectiveness of using 25% dextrose injection compared with corticosteroid injection into the facet joint for the treatment of CLBP [61]. The study included 178 patients with CLBP without indication for surgical intervention. Among them, 91 patients received facet joint injection with corticosteroids and 87 underwent prolotherapy. The group of patients with corticosteroid administration received 20 mg of methylprednisolone combined with 2–4 mL of 0.25% bupivacaine. The prolotherapy group received 5 mL of 25% dextrose [61]. Both groups were evaluated using VAS for pain and the ODI for functional status. Measurements were recorded at baseline, one day after the procedure, on the 15th day after the procedure, and at three months post-injection [61]. At baseline, patients in the corticosteroid group reported significantly higher VAS scores than those in the prolotherapy group. However, one day after the intervention, the corticosteroid group had a significant improvement in pain compared with the prolotherapy group. By day 15, pain levels were similar in both groups, with no statistically significant difference. At the three-month follow-up, patients from the prolotherapy group had a significant improvement regarding the pain, with significantly lower VAS scores than those from the corticosteroid group [61]. In terms of functional improvement, ODI scores were comparable between groups before treatment. At the three-month evaluation, the corticosteroid group showed better ODI outcomes, suggesting greater improvement in physical function compared to the prolotherapy group [61]. These findings suggest that while corticosteroids may offer quicker short-term pain relief, prolotherapy appears to provide more sustained analgesic effects in the long term, as reflected by more favorable VAS scores. Additionally, facet joint injections demonstrated greater improvement in physical function at the three-month mark, as indicated by better ODI scores compared to prolotherapy [61].

Gholamreza Raissi et al. (2022) [62] conducted a randomized, double-blind study involving 36 patients diagnosed with sacroiliac joint dysfunction (SIJD). The diagnosis was based on clinical symptoms, including pain in the hip, thigh, and groin regions, a positive Patrick’s or Gaenslen’s test, and tenderness below the postero-superior iliac spine. All participants had previously shown no improvement with pharmacological treatment [62]. The patients were randomly enrolled into two equal groups. Each group received a single injection, either 2.5 mL of 20% dextrose solution or 2.5 mL of triamcinolone (40 mg/mL), both under ultrasound guidance, and after 2 mL of 2.5% intra-articular bupivacaine [62]. In addition, a stretching program and Acetaminophen were recommended to each patient to help prevent any post-injection side effects [62]. Pain intensity was measured using VAS, while functional status was evaluated using the DPQ, which captures the impact of chronic pain across daily activities, work, mood, and social engagement [62]. Patients were evaluated at 2, 8, and 36 weeks after the injection with VAS, and DPQ was evaluated up to 8 weeks. At baseline, there were no significant differences between groups regarding demographic or clinical variables, supporting the validity of between-group comparisons. Both groups had decreasing VAS scores from baseline, at 2, 8, and 36 weeks. Notably, the maximum therapeutic benefit in both groups occurred within the first two weeks, after which the scores remained constant. When comparing the scores of the groups, there were no statistically significant differences at any point in terms of VAS scores [62]. Analyzing functional improvement with the DPQ scale, at 2 and 8 weeks after the injection, only the corticosteroid group had statistically significant improvement in DPQ scores compared with baseline [62]. This study found that both prolotherapy and corticosteroid injections under ultrasound guidance led to a reduction in pain for patients with SIJD, while the corticosteroid group demonstrated slightly better short-term functional improvement [62].

In the observational retrospective study conducted by Carl PC Chen et al. (2023), two distinct ultrasound-guided interventional techniques were compared in the management of lumbar spinal stenosis (LSS) in elderly patients [63]. The authors aimed to evaluate the clinical efficacy of prolotherapy with 5% dextrose in water (D5W) injected into the multifidus muscle, versus mechanical needling combined with sterile water injection administered into multiple posterior lumbar structures, including facet joints, medial branches, and multifidus muscle [63]. There were 211 patients, divided into two groups. In the prolotherapy group were 107 patients who received a single session of ultrasound-guided injection of D5W into the multifidus muscle. In the other group, 104 patients were enrolled and they received a mechanical needling procedure with sterile water, and underwent four weekly sessions involving a more invasive protocol and technique. This technique utilized ultrasound guidance to insert a fine needle in proximity to the facet joints, medical branch, and multifidus muscle to mechanically disrupt fibrosis and calcifications commonly present in degenerative spinal changes. Following mechanical abrasion (needling), sterile water was injected into the treated zones to promote neurochemical modulation and potential modulation of central sensitization pathways [63]. Patients in both groups were evaluated at six distinct timepoints: baseline (T0), immediately post-intervention (T1), one week after (T2), one month after (T3), three months (T4), and six months after the procedure (T5). Primary outcomes included changes in pain intensity (regarding low back pain and radicular leg pain) measured using the visual analogue scale (VAS), and functional improvement assessed by walking distance until symptoms appeared [63]. The results revealed significant differences in therapeutic response between the two intervention groups. Patients who received ultrasound-guided mechanical needling combined with sterile water injections experienced a statistically significant reduction in LBP and radicular leg pain as measured by VAS, with improvements sustained from the first week (T1), through the 6-month follow-up (T5). Notably, this group also demonstrated progressive improvement in gait function, with walking distance increasing significantly at 1 (T3), 3 (T4), and 6 months post-treatment (T5) compared with baseline [63]. In contrast, patients treated with prolotherapy exhibited only moderate reductions in pain intensity, with statistically significant improvements in VAS scores for LBP and leg pain observed at T3 and T4. However, these effects declined by T5 follow-up, with no sustained benefit beyond the third month. Similarly, walking distance improved modestly in the prolotherapy group at T3 and T4 follow-up, but the gains were not maintained long-term, and overall functional outcomes remained inferior to the mechanical needling group throughout the study period [63]. Furthermore, the authors reported a notably higher incidence of transient adverse effects such as injection site pain, dizziness, and leg numbness in the prolotherapy group. In contrast, the mechanical needling group had fewer and less intense post-procedural symptoms [63]. Taken together, the findings suggest that while both interventions provided some degree of short-term symptomatic relief, the mechanical needling and sterile water approach demonstrated superior and longer-lasting efficacy in reducing axial and radicular pain, as well as enhancing ambulatory function, in older patients with lumbar spinal stenosis [63].

The clinical trial conducted by Pereira Pires et al. (2023) [64] aimed to evaluate the efficacy of 75% hypertonic glucose injections compared to conservative treatment in patients with CLBP. The study included 38 patients, randomized equally into two groups: one receiving facet joint infiltration with glucose and lifestyle modifications, and the other undergoing conventional conservative management, including lifestyle and behavioral interventions. The intervention group received a single injection of 1 mL/kg of 75% hypertonic glucose directly into the facet joints (L1-L2, L5-S1) [64]. Both groups were followed up at 1, 3, and 6 months using VAS and the Roland–Morris Disability Questionnaire (RMDQ). The authors did not specify whether the facet joint infiltrations were performed under ultrasound guidance. At 3 months, there was a statistically significant increase in VAS and Roland–Morris scores in the glucose group, but this did not persist at 6-month follow-up evaluation. The study found no significant long-term differences between the prolotherapy and conservative groups. Both groups showed improvement compared to baseline, but the authors concluded that the prolotherapy protocol showed no added value over conservative care in this study [64].

Yasmine Ragab Elsayed Mohamed et al. (2024) conducted a prospective, randomized, double-blind clinical trial to compare the effectiveness of caudal epidural prolotherapy versus corticosteroid injection for the treatment of CLBP in patients with failed back surgery syndrome (FBSS) [65]. A total of 90 participants with FBSS and chronic pain lasting more than three months were recruited and randomly assigned into two equal groups: one group received caudal epidural prolotherapy (dextrose prolotherapy), and the other received methylprednisolone-based steroid injection. All injections were administered using combined ultrasound and fluoroscopy guidance, ensuring anatomical precision and procedural consistency. The prolotherapy group received 10 mL of 25% dextrose, 5 mL of 0.5% bupivacaine, 4 mL of contrast agent, and 6 mL of distilled water. The steroid group received 1 mL of methylprednisolone (40 mg/mL), 5 mL of 0.5% bupivacaine, 4 mL of contrast, and 15 mL of distilled water [65]. Both groups underwent a single injection, with follow-up assessments at 2, 4, 6, and 8 weeks and at 3 and 6 months. Primary outcome measures included the VAS scale for pain, while secondary outcomes were assessed via ODI and MPQ [65]. Both groups showed statistically significant improvement in pain, function, and quality of life at follow-up points. However, no significant difference was observed between groups during the early post-injection phase (2–6 weeks). Starting at 8 weeks and extending through 3 and 6 months, the steroid group had significantly lower VAS, ODI, and MPQ scores than the prolotherapy group, suggesting a longer-lasting analgesic effect [65]. While both interventions produced significant clinical improvement, steroid injections were more effective in maintaining long-term pain relief and functional improvement over six months. However, this finding must be interpreted in the context of a single-session prolotherapy protocol, which may not reflect its full regenerative potential [65]. 

**Table 2:** Summary of included studies.

Authors and Publication Year/Country	Pathology	Study Design	Sample Size	Intervention Protocols	Outcome Measures Related to Pain Reduction and Lumbar Function	No of Reff.
Liza Maniquis-Smigel et al., 2017 [59] Unites States of America	CLBP with radiation to buttock/leg, including -Lumbar spinal stenosis (34%) -Lumbar radiculopathy (26%) -Nonspecific low back pain (26%) -Peripheral neuropathy (6%) -Failed back surgery (11%)	Randomized Double-Blind Controlled Trial	Of 56 eligible patients, 19 declined and 2 were excluded (due to substantial cramping pain before receiving the full 10 mL injection), leaving 35 participants (19 dextrose, 16 saline).	Intervention: Single caudal epidural injection of 10 mL 5% dextrose. Control: Single caudal epidural injection of 10 mL 0.9% saline. Technique: Vertical caudal approach under epidurography guidance (25 G needle).	Outcome measures: -Change in a numerical rating scale (NRS) pain score (0–10). Follow-up: Assessments at 15 min, 2 h, 4 h, 48 h, 2 weeks post-injection. Key Findings: -Epidural 5% dextrose is a rapid-acting, safe neurogenic analgesic for short-term CLBP management.	50
Özlem Köroğlu et al., 2019 [60] Turkey	Radicular Low Back Pain due to lumbar disc herniation	Retrospective study	Total: 40 patients 20 participants: Prolotherapy only, 20 participants: Prolotherapy + Physical Therapy.	-5% Dextrose prolotherapy injections at iliolumbar/transverse ligament insertions and facet joints -3 injection sessions at 4-week intervals Group 1: Prolotherapy only. Group 2: Prolotherapy + Physical Therapy: Transcutaneous Electrical Nerve Stimulation (TENS), infrared, stretching exercises; 15 sessions. -NSAIDs prohibited (only paracetamol allowed).	Outcome measures: -Visual Analog Scale (VAS) for pain intensity. -Oswestry Disability Index (ODI) for functional disability. -36-Item Short Form Survey (SF-36) for quality of life. Follow-up: Assessments at baseline, 3, 12, and 52 weeks. Key Findings: -5% dextrose prolotherapy significantly reduces pain and disability in chronic radicular low back pain from lumbar disc herniation, with effects lasting up to 1 year. -Adding physical therapy did not enhance outcomes, supporting prolotherapy as a practical primary treatment.	21
Timur Yildirim et al., 2021 [61] Turkey	CLBP (mechanical origin)	Retrospective Comparative Study	Total: 178 patients Group 1: 91 participants—Facet joint injection Group 2: 87 participants—Prolotherapy	Facet Joint Injection: 20 mg methylprednisolone + 2–4 mL 0.25% bupivacaine per single-level facet joint. Prolotherapy: 5 mL 25% dextrose solution injected into the facet joint capsule per single level.	Outcome measures: -Visual Analog Scale (VAS) measured at baseline, day 1, day 15, and month 3. -Lumbar Function: Oswestry Disability Index (ODI) was measured at baseline and month 3. Key Findings: -Facet injection reduced VAS more effectively at day 1 (*p* < 0.001). -Prolotherapy showed superior VAS reduction at month 3 (*p* < 0.001). -ODI scores were higher (worse function) in the prolotherapy group than the corticosteroid group at month 3 (*p* < 0.001).	15
Gholamreza Raissi et al., 2022 [62] Iran	Sacroiliac joint dysfunction (SIJD) as an etiology of low back pain (LBP)	Randomized double-blind clinical trial (RCT)	Total: 40 patients, 2 patients withdrew from each group for personal reasons Group 1:18 participants—dextrose prolotherapy; Group 2: 18 participants—corticosteroid.	Prolotherapy Group: Single ultrasound-guided injection of 2.5 mL 20% dextrose solution. Corticosteroid Group: Single ultrasound-guided injection of 2.5 mL triamcinolone (40 mg/mL). Technique: -Approach: Inferomedial (1 inch medial and below Posterior Superior Iliac Spine); -Following the intra-articular injection of 2 mL of 2.5% bupivacaine; -Guidance: Real-time ultrasound (transverse to sacral hiatus); -Needle: 22-gauge spinal needle.	Outcome measures: -Visual Analog Scale (VAS) measured at Baseline, 2 weeks, 8 weeks, and 36 weeks; -Dallas Pain Questionnaire (DPQ) measured at Baseline, 2 weeks, and 8 weeks. Key Findings: VAS: -Significant pain reduction in both groups at all timepoints, no difference between groups; DPQ: -Corticosteroid group: Significant functional improvement at 2 and 8 weeks; -Dextrose group: Non-significant improvement.	20
Carl PC Chen et al., 2023 [63] China	Lumbar Spinal Stenosis (LSS)	Observational Retrospective	Total: 211 patients Group 1: 104 participants— mechanical needling + sterile water; Group 2: 107 participants— polotherapy: Dextrose 5% in Water (D5W).	Group 1: ultrasound-guided (USG) mechanical needling + sterile water injection into facet joints, medial branches, and multifidus muscles (4 weekly sessions; 1 mL/site, 3 mL/level, total 12 mL/session); Group 2: Single USG injection of 5% dextrose water into the multifidus muscles.	Outcome measures: -VAS for low back pain (0–10); -VAS for leg/radicular pain (0–10); -Walking distance (meters before calf pain). Follow-up: Assessments at Pre-injection, immediately post-injection, 1 week, 1 month, 3 months, 6 months. Key Findings: -Mechanical needling + sterile water: Significantly reduced back/leg pain (VAS) and increased walking distance vs. baseline at 1, 3, and 6 months; -Prolotherapy (D5W): Moderate pain reduction and improved walking distance only at 1 week and 1 month. Effects diminished by 3 months (VAS/walking distance reverted to baseline).	20
Jose Alberto Pereira Pires et al., 2023 [64] Brazil	CLBP, non-traumatic, unresponsive to physical therapy. Facet joint degeneration confirmed.	Randomized, blinded clinical trial.	Total: 40 patients, 2 lost to follow-up Group 1: 19 participants—hypertonic glucose Group 2: 19 participants—conservative group	Hypertonic glucose Group: Single facet joint injection of 75% hypertonic glucose (1 mL/kg) + lifestyle modifications; Conservative Group: Clinical management (diet/lifestyle changes) only.	Outcome measures: -Visual Analog Scale (VAS) for pain reduction, -Roland–Morris Disability Questionnaire for lumbar function. Follow-up: assessments at 1, 3, and 6 months. Key Findings: -Both groups showed significant improvement in VAS and Roland–Morris scores in dynamics; -The authors concluded that prolotherapy did not outperform conservative care.	37
Yasmine Ragab Elsayed Mohamed et al., 2024 [65] Egypt	Failed Back Surgery Syndrome (FBSS) with CLBP	Prospective, randomized, double-blinded clinical trial	Total: 90 patients, 7 lost to follow-up Group 1: 40 participants—Prolotherapy; Group 2: 43 participants—Steroid.	Prolotherapy Group: US + fluoroscopy-guided caudal epidural injection of: -5 mL bupivacaine 0.5%; -4 mL Omnipaque contrast (350 mg/mL); -10 mL dextrose 25%; -6 mL distilled water (Total volume: 25 mL); Steroid Group: US + fluoroscopy-guided caudal epidural injection of: -1 mL methylprednisolone (40 mg/mL); -4 mL Omnipaque contrast (350 mg/mL); -5 mL bupivacaine 0.5%; -15 mL distilled water (Total volume: 25 mL).	Outcome measures: -Visual Analog Scale (VAS) for pain; -Oswestry Disability Index (ODI); -McGill Pain Questionnaire (MPQ); Follow-up: Assessments at 2, 4, 6, 8 weeks, 3 and 6 months post-injection. Key Findings: -No significant difference was observed between groups during the early post-injection phase (2–6 weeks); -Steroids provide superior long-term relief ( > 6 weeks); -Both have comparable safety.	13

Abbreviations: CLBP—Chronic low back pain; D5W—Dextrose 5% in Water; DPQ—Dallas Pain Questionnaire; FBSS—Failed Back Surgery Syndrome; LSS—Lumbar Spinal Stenosis; mL—milliliter; MPQ—McGill Pain Questionnaire; NRS—numerical rating scale; NSAIDs—non-steroidal anti-inflammatory drugs; ODI—Oswestry Disability Index; RCT—Randomized double-blind clinical trial; SF-36—36-Item Short Form Survey; SIJD—Sacroiliac joint dysfunction; TENS—Transcutaneous Electrical Nerve Stimulation; USA—The United States of America; USG—Ultrasound-guided; US—Ultrasound; VAS—Visual Analog Scale. No—number; Reff—References.

### 3.2. Structured Synthesis of Results

#### 3.2.1. Targeted Pathologies

**Figure 2:**
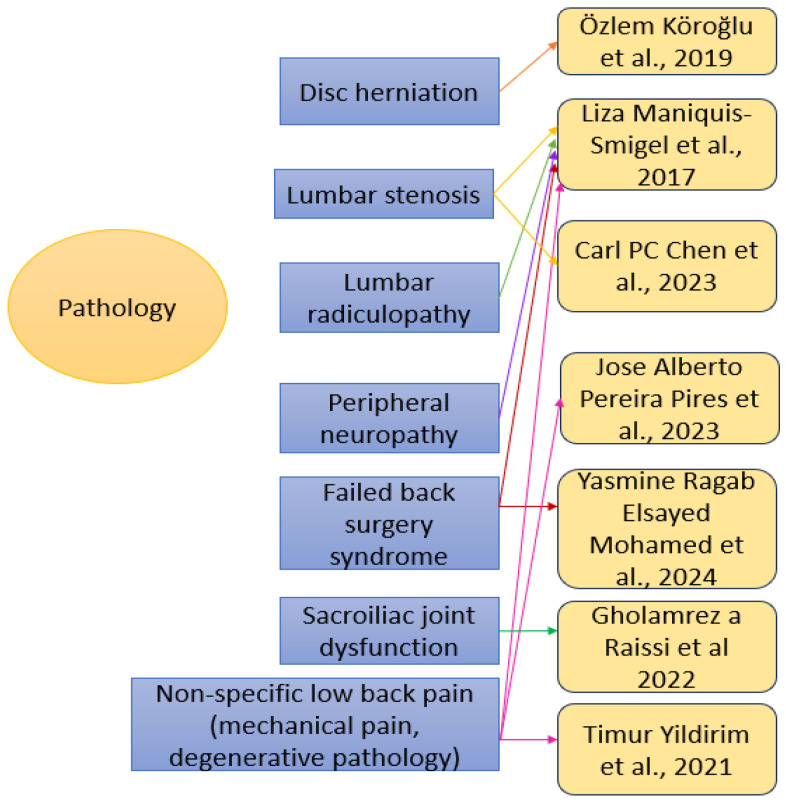
Tageted pathology from analyzed studies [59,60,61,62,63,64,65].

The included studies targeted various etiologies of chronic low back pain, such as the following (Figure 2): disc herniation—one study (Köroğlu et al. (2019) [60], lumbar stenosis—two studies (Maniquis-Smigel et al. (2017) [59]) and Chen et al. (2023) [63]), lumbar radiculopathy—one study (Maniquis-Smigel et al. (2017) [59]), peripheral neuropathy—one study (Maniquis-Smigel et al. (2017) [59]), failed back surgery syndrome—two studies (Maniquis- Smigel et al. (2017) [59] and Mohamed et al. (2024) [65]), sacroiliac joint dysfunction—one study (Raissi et al. (2022) [62]), and non-specific low back pain including mechanical pain and degenerative pathology—three studies (Maniquis -Smigel et al. (2017) [59], Pires et al. (2023) [64] and Yildirim et al. (2021) [61]).

#### 3.2.2. Control Group

**Figure 3:**
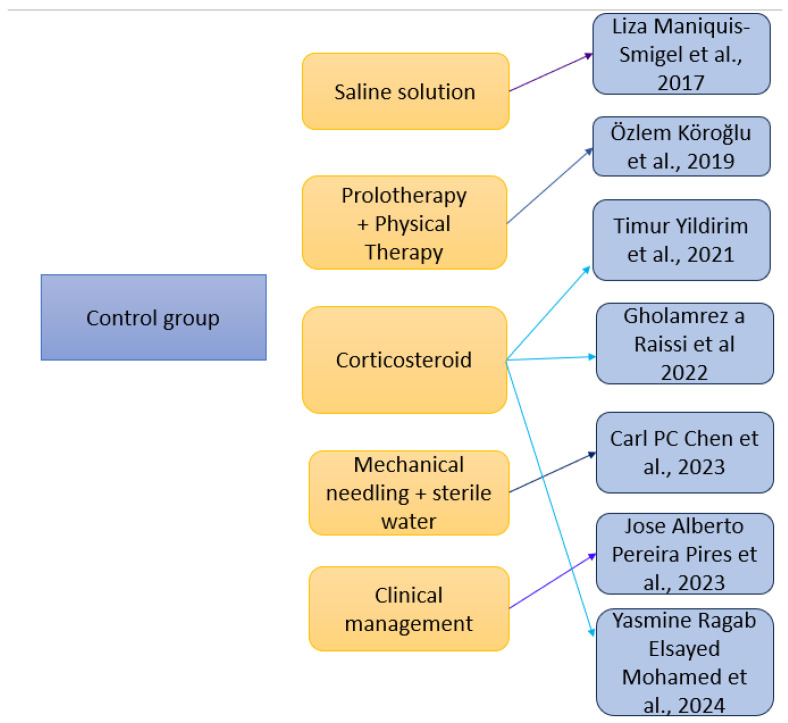
Control group used in analyzed studies [59,60,61,62,63,64,65].

Control conditions differed from one study to another (Figure 3) as following: one study used the saline solution (Maniquis-Smigel et al. (2017) [59]), prolotherapy (dextrose prolotherapy) and physical therapy were used also in one study (Köroğlu et al. (2019) [60]), corticosteroids were used in three studies (Yildirim et al. (2021) [61]), (Raissi et al. (2022) [62] and Mohamed et al. (2024) [65]), mechanical needling and sterile water was used in one study (Chen et al. (2023) [63]), and clinical management such as diet and lifestyle changes was used in one study (Pires et al. (2023) [64]).

#### 3.2.3. Dextrose Prolotherapy Concentration and Injection Protocols

**Figure 4:**
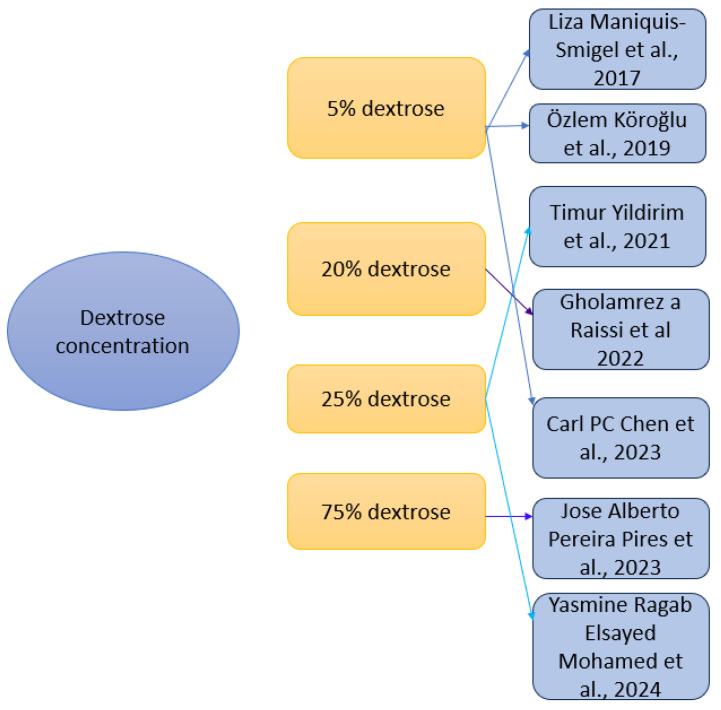
Dextrose prolotherapy concentration used in analyzed studies [59,60,61,62,63,64,65].

The studies used different dextrose concentrations (Figure 4) and administration techniques: 5% dextrose concentration was most frequently used—in three studies (Maniquis-Smigel et al. (2017 [59], Köroğlu et al. (2019) [60] and Chen et al. (2023) [63]), administrated via caudal epidural, spinal, or periarticular injections; 20% dextrose concentration was used in one study (Raissi et al. (2022) [62]) in sacroiliac joint injection; 25% dextrose concentration was used in two studies (Yildirim et al. (2021) [61] and Mohamed et al. (2024) [65]) and was administered into facet joints or via the caudal approach; and 75% dextrose concentration was used in one study (Pires et al. (2023) [64]), administered into facet joints. Protocols varied from single injection intervention, which was used in six studies [59,61,62,63,64,65], or three injection sessions at 4-week intervals, used in one study [60]. Image guidance included ultrasound [62,63], fluoroscopy [64] or both techniques [65]. Other studies used anatomical landmarks [59,60,61].

#### 3.2.4. Outcome Measures

**Figure 5:**
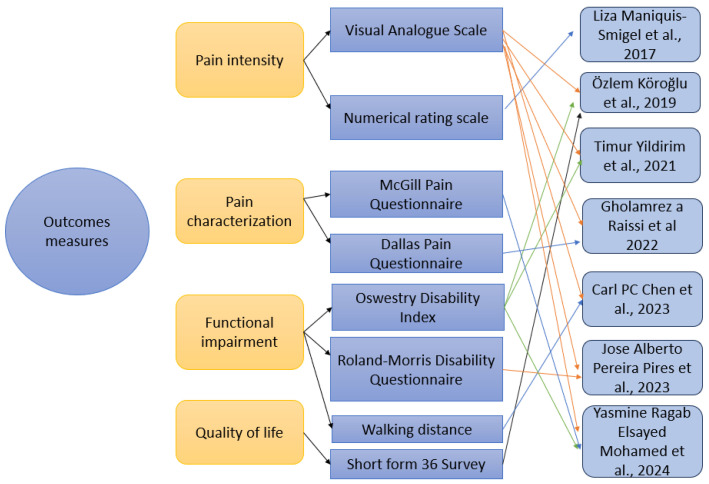
Outcome measures used in analyzed studies [59,60,61,62,63,64,65].

Pain intensity and functionality (Figure 5) were the most frequently assessed outcomes. Pain was measured with two scales—Visual Analogue Scale (VAS) used by six studies [60,61,62,63,64,65] and Numeric Rating Scale (NRS) used by one study [59]. Also, for pain, two questionnaires were used—the McGill Pain Questionnaire (MPQ), used for pain characterization, found in one study [65], and the Dallas Pain Questionnaire (DPQ), used for measuring how pain impacts the daily activities, used in one study [62]. For functional impairment, the Oswestry Disability Index (ODI) was used in three studies [60,61,65], and the Roland–Morris Disability Questionnaire (RMDQ) was used in one study [64]. Walking distance was measured by a single study [63]. Quality of life of patients with CLBP was measured using Short Form 36 Questionnaire (SF-36) and only one study used this scale [60].

#### 3.2.5. Follow-Up Duration

**Figure 6:**
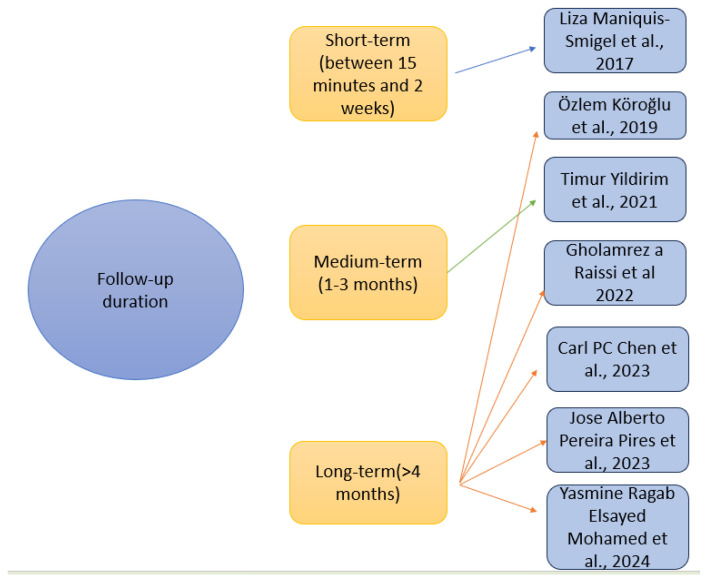
Follow-up duration used in the analyzed studies [59,60,61,62,63,64,65].

The duration of follow-up assessments varied across studies, ranging from short-term evaluations to long-term outcome measurements (Figure 6). Only Maniquis-Smigel et al. (2017) used a short-term follow-up for the outcomes used, from 15 min up to two weeks [59]. Yildirim et al. (2021) used a medium-term follow-up from day one up to 3 months [61]. Long-term was used by the other studies, as follows: Köroğlu et al. (2019) used a follow-up term from 3 to 52 weeks [60], Raissi et al. (2022) used at baseline up to 36 weeks [62], Chen et al. (2023), Pires et al. (2023), and Mohamed et al. (2024) used immediately after the intervention and up to 6 months [63,64,65].

### 3.3. Risk Bias

**Figure 7:**
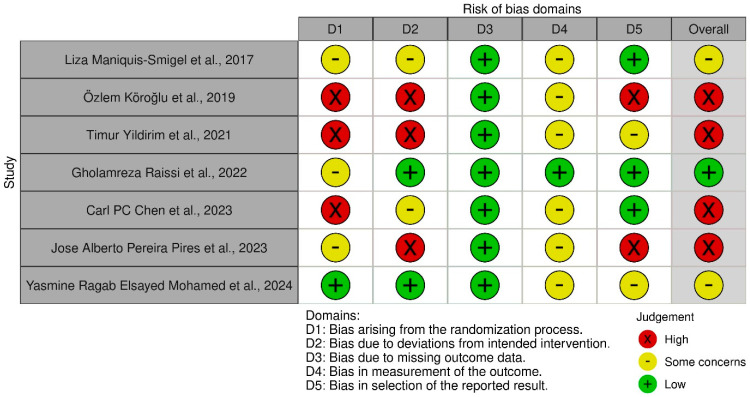
Risk of bias assessment using Cochrane ROB-2 for the included studies [59,60,61,62,63,64,65].

**Figure 8:**
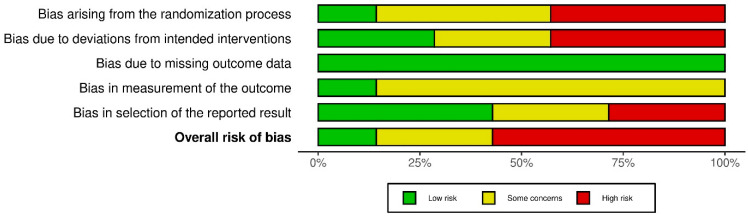
Summary plot of Cochrane ROB-2 for the included studies.

The Cochrane ROB-2 tool assesses risk of bias in randomized trials across five domains, including the randomization process, deviations from intended interventions, missing outcome data, measurement of the outcome, and selection of the reported result [66]. The tool was applied to the seven included studies, each of which was systematically assessed across all domains and assigned a rating of low risk, some concerns, or high risk of bias. Overall, the majority of studies were found to have either a high risk of bias or raised some concerns (Figure 7 and Figure 8).

For a more detailed analysis of bias, the PEDro scale was used. The PEDro scale’s 10 items provide a valid and comprehensive assessment of methodological quality in clinical trials, covering key aspects such as eligibility criteria, randomization, blinding, follow-up, and statistical reporting, which evaluate potential biases and the rigor of trial conduct [67,68].

The seven studies included in this analysis demonstrated a wide range of methodological quality, with PEDro scores varying from poor (3/10) to excellent (9/10). The variability observed highlighted the importance of critically appraising the studies to ensure accurate interpretation of findings and sound conclusions (Table 3).

## 4. Discussion

Although prolotherapy (dextrose prolotherapy) has drawn considerable attention as a regenerative treatment, its clinical effectiveness remains controversial in the context of CLBP. This systematic review aims, after analyzing the included studies, to discuss key variables such as concentration, injection volumes and solution composition, injection sites and anatomical targets, imaging guidance, number of injection sessions, and the variability of control groups.

### 4.1. Diversity of Methodology (Substances, Concentration)

Dextrose prolotherapy has been explored as a regenerative, minimally invasive intervention for CLBP. Analyzing the seven clinical studies from our review, they highlight substantial heterogeneity in clinical application, particularly in the choice of concentration, ranging from 5% to 75%. Liza Maniquis-Smigel et al., 2017 [59], Özlem Köroğlu et al., 2019 [60], and Carl PC Chen et al., 2023 [63] utilized in their study a 5% concentration of dextrose, Gholamreza Raissi et al., 2022 [62] a 20% concentration, while Timur Yildirim et al., 2021 [61] and Yasmine Ragab Elsayed Mohamed et al., 2024 [65] utilized a 25% concentration, and only Jose Alberto Pereira Pires et al., 2023 [64] utilized a 75% concentration. There is a notable variability in the concentrations of dextrose used for CLBP.

In the scientific literature, published studies have most commonly used concentrations between 5% and 50% dextrose for low back pain [52,69], and higher concentrations, like 75% hypertonic glucose, are not mentioned in other studies published in the literature. However, there is currently no consensus on the optimal concentration that should be used for effective and safe treatment. The absence of standardized protocols makes it difficult to compare results between studies and makes it challenging to apply the findings to general clinical practice.

Analyzing our selected studies, each concentration of dextrose appears to have a specific therapeutic effect, based on a particular mechanism of action.

Lower concentrations, such as 5% dextrose, appear to have neuromodulatory and mild proliferative mechanisms. In the study conducted by Liza Maniquis-Smigel et al., 2017 [59], 5% dextrose had a significant analgesic effect within 15 min, lasting up to 48 h. The authors attributed this effect to a direct neurogenic mechanism potentially involving transient receptor potential vanilloid 1 (TRPV1) channels at the dorsal root ganglion [59]. Similarly, Özlem Köroğlu et al., 2019 [60] also utilized 5% dextrose and provided a detailed mechanism, suggesting that dextrose induces mild osmotic stress, triggering a localized proliferative response, potentially via fibroblast stimulation and angiogenic gene expression [60]. The authors provide a clear explanation regarding the potential of dextrose to stimulate healing via inflammatory cytokine release and mild tissue dehydration. On the other hand, Carl P.C. Chen et al., 2023 [63], who also used 5% dextrose, did not provide any discussion of biological mechanisms.

Regarding the use of 20% dextrose concentration, Gholamreza Raissi et al., 2022 [62] did not provide an in-depth mechanistic analysis. This concentration is commonly associated with proliferative effects through inflammation-mediated regeneration [70].

Timur Yildirim et al., 2021 [61] provided a detailed biological rationale for using 25% dextrose, describing the mechanism as an inflammatory-proliferative process. In their explanation, the hyperosmolar solution induces localized inflammation, which subsequently activates fibroblasts and stimulates collagen production, ultimately promoting long-term tissue repair [61]. Yasmine Ragab Elsayed Mohamed et al., 2024 [65] also applied 25% dextrose, but they did not explicitly discuss the biological mechanism in their manuscript.

In the study conducted by Jose Alberto Pereira Pires et al., 2023 [64], the authors used 75% hypertonic glucose but did not provide any biological explanation for its use. However, it is established that such high concentrations of hypertonic glucose are used as a sclerosing agent in vascular sclerotherapy [71].

There is a substantial variability in biological mechanisms associated with different dextrose concentrations in prolotherapy for CLBP. While some studies describe these mechanisms, others do not address them at all. Further research is needed to compare the biological effects of varying dextrose concentrations using standardized protocols.

In addition to concentration, both the total volume of dextrose solution administered and its composition, whether combined with local anesthetics or used alone, may influence the clinical and biological outcomes of prolotherapy for CLBP. We observed variability in the parameters of the included studies.

For example, Liza Maniquis-Smigel et al., 2017 administered 10 mL of 5% dextrose without an anesthetic mixture [59]. Similarly, Özlem Köroğlu et al., 2019 and Carl P.C. Chen et al., 2023 injected 5 mL of 5% dextrose per session, also without co-injection of anesthetics [60,63]. Gholamreza Raissi et al., 2022 injected 2.5 mL of 20% dextrose also without anesthetic [62]. On the other hand, Yasmine Ragab Elsayed Mohamed et al., 2024 used 5 mL of 25% dextrose, diluted with 1 mL of lidocaine 2%, highlighting a combined analgesic and regenerative strategy [65]. Timur Yildirim et al., 2021 used 2 mL of 25% dextrose with no anesthetic mentioned in the protocol [61]. Jose Alberto Pereira Pires et al., 2023 administered 1 to 2 mL of 75% dextrose [64].

This wide range of injection volumes, from 2 mL to 10 mL per site, as well as the varying use of anesthetics, may significantly modulate the observed therapeutic responses. Larger injection volumes can potentially extend the area of tissue contact and modulate local pressure effects, while the inclusion of anesthetic may temporarily mask post-injection pain.

Importantly, these factors complicate direct comparisons between studies. Evaluating both the total dextrose dose and solution components is critical to accurately interpret clinical outcomes and mechanical implications across prolotherapy protocols.

### 4.2. Anatomical Approach

One critical variable in prolotherapy (dextrose prolotherapy) research for CLBP is the specific anatomical site of injection. The review targeted different structures, reflecting the complex and multifactorial nature of CLBP, which encompasses various pain generators, such as the intervertebral disc, facet joints, ligaments, sacroiliac joint, nerve roots, and paraspinal muscles.

Liza Maniquis-Smigel et al., 2017 performed epidural injections, providing dextrose into the epidural space with the primary goal of targeting nerve roots and perineural tissues [59]. Özlem Köroğlu et al., 2019 injected near the paraspinal ligaments and nerve roots for radicular low back pain, emphasizing neural involvement [60]. Timur Yildirim et al., 2021 targeted facet joints, a common source of axial low back pain associated with arthropathy [61]. Gholamreza Raissi et al., 2022 administered dextrose into the sacroiliac joint, addressing pain linked to pelvic instability or sacroiliac dysfunction [62]. Carl PC Chen et al., 2023 administered injections directly into the multifidus muscle, a deep paraspinal stabilizer implicated in muscular dysfunction in CLBP [63]. Jose Alberto Pereira Pires et al., 2023 injected directly into lumbar facet joints, addressing facet joint pain [64]. Yasmine Ragab Elsayed Mohamed et al., 2024 used caudal epidural injections, again focusing on the epidural space and nerve root involvement [65].

This diversity of injection sites underscores the variability of CLBP as a clinical entity. Unlike focal disorders with a single pain generator, CLBP involves overlapping anatomical, biomechanical, and neurogenic components, including discogenic pain, facet joint arthropathy, sacroiliac joint dysfunction, and myofascial sources of pain.

Therefore, comparing the outcomes across these studies requires caution. Not only do injection volumes and solution compositions vary, but also the targeted anatomical structures differ considerably, reflecting distinct pathophysiological mechanisms. This reinforces the need for individualized treatment approaches in CLBP.

### 4.3. Using Imaging Guidance

The use of imaging guidance during prolotherapy (dextrose prolotherapy) injections represents another crucial methodological variable across studies investigating CLBP. The presence or absence of imaging guidance, as well as the type of imaging modality employed, can significantly impact both the accuracy of injection delivery and clinical outcomes.

Studies using imaging guidance: Carl PC Chen et al., 2023 used ultrasound guidance for injections into the multifidus muscle [63]. Yasmine Ragab Elsayed Mohamed et al., 2024 used ultrasound and fluoroscopy guidance for caudal epidural injections, aiming to enhance safety and accuracy in accessing the epidural space [65]. Gholamreza Raissi et al., 2022 used ultrasound guidance for sacroiliac joint injections, targeting a deep joint [62]. Jose Alberto Pereira Pires et al., 2023 performed fluoroscopy-guided injections into the lumbar facet joints [64].

Studies without imaging guidance: Liza Maniquis-Smigel et al., 2017 performed epidural injections without imaging guidance, using anatomical landmarks for needle placement [59]. Özlem Köroğlu et al., 2019 administered injections using anatomical landmarks without imaging, targeting paraspinal regions and nerve root areas [60]. Timur Yildirim et al., 2021 conducted facet joint injections without imaging guidance, relying on palpation and anatomical landmarks [61].

This clear distinction between image-guided and non-guided techniques is important for interpreting clinical results. Imaging guidance, particularly with ultrasound or fluoroscopy, or using both, increases injection precision, especially for deep or complex anatomical targets such as the epidural space, sacroiliac joints, or facet joints. Studies without imaging guidance carry a higher risk of misplacement or suboptimal delivery, potentially affecting both efficacy and safety outcomes. Moreover, imaging-guided procedures often require greater technical expertise and resources. The variation in injection techniques among these studies complicates direct comparison of outcomes and underscores the need for standardized reporting of guidance methods in future prolotherapy trials for CLBP.

### 4.4. Number of Sessions for Injection

Another methodological aspect that varies widely across prolotherapy studies for CLBP is the number of injection sessions administered. The therapeutic response to prolotherapy may depend not only on the concentration and volume of dextrose but also on the frequency and repetition of treatments, which can significantly influence tissue remodeling and long-term outcomes.

From the analyzed studies, there were four studies (Liza Maniquis-Smigel et al., 2017; Yasmine Ragab Elsayed Mohamed et al., 2024; Gholamreza Raissi et al., 2022; and Jose Alberto Pereira Pires et al., 2023) that used a single injection session in their study groups of patients [59,62,64,65]. On the other hand, there were studies with multiple injection sessions, such as Özlem Köroğlu et al., 2019—administered three sessions of 5% dextrose at two-week intervals, Carl PC Chen et al., 2023—provided multiple injection sessions targeting the multifidus muscle, once a week, for four weeks, and Timur Yildirim et al., 2021—performed three injection sessions into the facet joints, with injections spaced one week apart [60,61,63].

This variability in injection frequency complicated the comparison of outcomes across studies. While some protocols rely on a single injection aimed at achieving immediate or short-term relief, others repeat the treatment to promote cumulative regenerative effects and longer-lasting clinical benefits. Notably, prolotherapy (dextrose prolotherapy) protocols with multiple sessions may be more aligned with the theoretical regenerative mechanism of action, as fibroblast activation, collagen synthesis, and tissue remodeling are typically gradual processes requiring repeated stimulation over time. Therefore, future research should aim to systematically evaluate the dose–response relationship not only in terms of concentration and volume but also in terms of number and timing of injection sessions, to optimize clinical protocols for CLBP.

Control groups utilized in prolotherapy (dextrose prolotherapy) trials for CLBP represent an important methodological factor that significantly influences the interpretation of clinical efficacy. Across the reviewed studies, the types of control groups varied widely, including corticosteroid injections, placebo interventions such as saline or mechanical needling, and even standard conservative care. This variability introduces challenges when attempting to compare outcomes across trials.

Studies using corticosteroid injections for the control group: Timur Yildirim et al., 2021 used 20 mg methylprednisolone and 2–4 mL 0.25% bupivacaine, while Gholamreza Raissi et al., 2022 injected 2.5 mL triamcinolone (40 mg/mL), and Yasmine Ragab Elsayed Mohamed et al., 2024 used 1 mL methylprednisolone (40 mg/mL) with 5 mL bupivacaine 0.5% [61,62,65].

Findings from the included studies indicate that in the short term, corticosteroids provide faster pain relief and slightly better functional improvement, while in the long term, prolotherapy offers significantly more sustained analgesia [61], with both treatments potentially improving function and quality of life [65]. These results support other studies, which highlight that, in various pathologies in general, while steroids provide only short-term improvement, the prolotherapy group experiences lasting benefits beyond 3 months [72,73].

Studies using saline injections: Liza Maniquis-Smigel et al., 2017 used 10 mL 0.9% saline solution, while Carl PC Chen et al., 2023 used mechanical needling and sterile water injection [59,63].

Notably, Carl PC Chen et al., 2023 demonstrated that the mechanical needling and sterile water approach provides superior and longer-lasting effectiveness in reducing both axial and radicular pain, as well as enhancing ambulatory function in older patients with lumbar spinal stenosis [63]. Also, another study supports that USG mechanical needling with sterile water injections at the lumbar facet joints, medial branch of the facet joint, and multifidus muscles can relieve pain for a minimum of 6 months [74]. Taken together, the available evidence indicates that both prolotherapy and mechanical needling with sterile water may confer benefit in selected cases of CLBP.

### 4.5. Control Groups

On the other hand, there were studies with different control groups, such as Özlem Köroğlu et al., 2019, in which the control group was Prolotherapy + Physical Therapy (TENS, infrared, stretching exercises), and Pereira Pires et al., 2023 used clinical management (diet/lifestyle changes) only [60,64].

There is limited evidence suggesting that dextrose-based prolotherapy offers a more effective therapeutic outcome than exercise [55]. Özlem Köroğlu et al., 2019 sustain that physical therapy did not enhance the effects of prolotherapy within this specific protocol [60]. It is important to emphasize that the rehabilitation approaches used were limited and that, in low back pain, treatment methods may be more varied.

Contrasting evidence exists; Yelland et al. found that the overall benefit of prolotherapy in non-specific CLBP was not superior to placebo or exercise therapy unless combined with adjunctive modalities such as spinal manipulation or rehabilitation programs [70].

This wide variation in control groups presents a major challenge for interpreting and comparing outcomes across studies. Trials using corticosteroids primarily examine differences between regenerative and anti-inflammatory therapies, while those using saline or mechanical interventions aim to separate the specific therapeutic effects of prolotherapy beyond placebo. Future research should prioritize rigorous selection of both active and placebo controls to improve methodological consistency and allow more meaningful comparisons between prolotherapy protocols in CLBP.

### 4.6. Scale-Based Assessment

The outcome measures utilized in the studies included in this systematic review comprised subjective tools such as the Numerical Rating Scale (NRS) and Visual Analog Scale (VAS) for pain assessment, applied at various time points ranging from minutes post-injection up to 52 weeks. They also included the Oswestry Disability Index (ODI) and Roland–Morris Disability Questionnaire (RMDQ) for evaluating functional disability, the Dallas Pain Questionnaire (DPQ) for assessing pain-related impact on daily activities, the 36-Item Short Form Survey (SF-36) for quality of life, the McGill Pain Questionnaire (MPQ) for detailed pain characterization, as well as objective measures like walking distance before calf pain. Given the predominantly subjective nature of these assessments, there remains a clear need for more objective and standardized methods to evaluate both pain and functional outcomes in future research.

### 4.7. Clinical Significance

Across the reviewed studies, dextrose prolotherapy consistently showed statistically significant improvements in pain scores, but the magnitude and duration of benefit varied considerably. Some studies reported substantial short-term pain reduction (for example over 50% decrease of NRS) such as the analgesic effect seen in Maniquis-Smigel et al. (2017), where 84% of patients receiving dextrose prolotherapy achieved over 50% pain reduction within 4 h after the intervention [59]. Short-term pain relief was obtained up to one month by Chen et al. (2023), and up to three months by Yildirim et al. (2021) and also by Raissi et al. (2022) [61,62,63]. Long-term improvements were documented in Köroğlu et al. (2019), where benefits in both pain and function persisted up to 12 months following three dextrose prolotherapy sessions [60]. In contrast, Pires et al. (2023) [64] used 75% glucose concentration for dextrose prolotherapy in a single-session protocol and found no additional benefit at 3 months, suggesting a limited clinical effect. Although these outcomes indicate potential efficacy, none of the studies provide standardized effect size measures, which limits direct comparison or quantification of clinical impact.

### 4.8. Functional Outcomes Versus Pain Outcomes

Although pain intensity (measured using VAS or NRS) was reported in all studies, functional improvement was less consistently assessed or observed. In some studies (Yildirim et al. (2021), Raissi et al. (2022)), pain reduction did not align with functional gains, as indicated by unchanged ODI scores, or with DPQ scores [61,62]. On the other hand, Köroğlu et al. (2019) and Mohamed et al. (2024) observed parallel improvements in both domains over long-term follow-up, suggesting that multi-session protocols or a targeted epidural approach may exert broader functional benefits [60,65]. Chen et al. (2023) assessed walking distance as a functional metric outcome and found greater gains in the mechanical needling group versus dextrose prolotherapy [63], reinforcing that pain relief does not necessarily translate into improved mobility. This discrepancy between symptom and function highlights the need for using an objective, rather than subjective, pain assessment tool and multidimensional outcome sets in future studies, ideally including objective physical performance measures in addition to self-reported scales.

### 4.9. Impact of Dextrose Concentration

The studies included a wide range of dextrose concentrations, from 5% (Smigel et al., Köroğlu et al., Chen et al.) [59,60,63], to 20% (Raissi et al.) [62], 25% (Yildirim et al., and Mohamed et al.) [61,65], and up to 75% (Pires et al.) [64]. Interestingly, outcomes did not appear to follow a linear dose–response trend. Studies using 5% dextrose reported both immediate [59] and sustained analgesic benefits [60], particularly when combined with repeated administration. Mid-range concentrations (20–25%) had mixed results—Yildirim et al. observed superior pain control at 3 months compared to corticosteroids [61], while Mohamed et al. found better outcomes in the long-term steroid group [65]. The use of 75% dextrose by Pires et al. [64] did not improve outcomes over conservative care, suggesting that extremely high concentrations may not enhance efficacy. These findings support the hypothesis that optimal therapeutic concentration depends not solely on concentration but also on the pathophysiology targeted, injection site, and a standardized protocol.

### 4.10. Anatomical Target and Guidance Modality

Differences in anatomical target and injection precision may partly explain the heterogeneity in outcomes. Studies targeting deep stabilizing or pain-generating structures (facet joint, sacroiliac joint, epidural space) under image guidance (Raissi et al., Chen et al., Mohamed et al.) tended to yield more consistent or sustained improvements [62,63,65]. Chen et al., who compared multifidus and facet joints prolotherapy versus mechanical needling, showed that targeting multiple structures under ultrasound guidance resulted in superior pain reduction and better functional outcomes [63]. In contrast, studies relying solely on anatomical landmarks (Yildirim et al., Köroğlu et al.) showed varied effects, potentially due to lower injection accuracy [60,61]. Furthermore, caudal epidural approaches (Maniquis-Smigel et al., Mohamed et al.) demonstrated rapid-onset pain relief, but their long-term effectiveness varied depending on guidance method and other substances used (local anesthetic or contrast substances) [59,65]. These results underline the importance of protocol standardization, particularly in relation to target specificity and the use of imaging for safe and controlled injection.

### 4.11. Summary of Outcomes According to Intervention Parameters

#### 4.11.1. Dextrose Concentration 

Dextrose concentration (Table 4) was variable: low concentration (5%) was associated with rapid-onset analgesia (Maniquis-Smigel et al., 2017) [59] and sustained improvements when administered in repeated sessions (Köroğlu et al., 2019) [61]; intermediate concentrations (20–25%) demonstrated variable findings: while Yildirim et al. (2021) reported superior pain relief at 3 months compared with corticosteroids at 25% concentration of dextrose [61], Mohamed et al. (2024) observed better outcomes in the corticosteroid group [65]; regarding high concentration (75%) as used by Pires et al. (2023) [64], this concentration did not demonstrate superiority over conservative care, indicating limited efficacy or a stagnation in treatment efficacy.

#### 4.11.2. Injection Frequency

Protocols involving multiple injection sessions (three sessions) were consistently linked to sustained improvements in pain and functional outcomes (Köroğlu et al., 2019) [61]. In contrast, single-injection strategies generally produced only short-term relief (Maniquis-Smigel et al., 2019, Yildirim et al., 2021, Chen et al., 2023) [59,61,63].

#### 4.11.3. Anatomical Target of Injection Appears to Play a Critical Role in Treatment Outcomes

Interventions directed at the facet joints (Yildirim et al., 2021) or sacroiliac joint (Raissi et al., 2022) were associated with favorable results, particularly in cases of mechanically mediated chronic low back pain [61,62]. Epidural injections tended to produce rapid symptomatic improvement, although their effects were on the short term (Maniquis-Smigel et al., 2017, Mohamed et al., 2024) [59,65]. In contrast, injections in multiple targets (facet joints, multifidus muscle—Chen et al., 2023) demonstrated modest improvements [63], but injections in multiple targets (iliolumbar/transverse ligament insertions and facet joint) with multi-session injections (Köroğlu et al., 2019) had improvement lasting up to 1 year [60].

#### 4.11.4. Guidance Modality of the Procedure Significantly Influenced Treatment Consistency and Effectiveness

Studies utilizing ultrasound and/or fluoroscopic guidance demonstrated more consistent and lasting improvements (Raissi et al., 2022, Chen et al., 2023, Mohamed et al., 2024), likely due to increased precision and safety [62,63,65]. In contrast, landmark-based techniques (Köroğlu et al., 2019, Yildirim et al., 2021) were associated with greater variability in outcomes, highlighting a potential limitation in accuracy [60,61].

**Table 4:** Outcomes according to intervention parameters.

Parameter	Category	Outcome Summary	Studies
Dextrose concentration	Low (5%)	Rapid analgesia, sustained effects with repeated sessions	Maniquis-Smigel et al., 2017 [59]; Köroğlu et al., 2019 [60]
	Intermediate (20–25%)	Variable outcomes: superior to corticosteroids at 25% concentration [61], versus inferior to corticosteroids at 20% concentration [65]	Yildirim et al., 2021 [61], Mohamed et al. [65]
	High (75%)	No added benefit over conservative care	Pires et al., 2023 [64]
Injection Frequency	Single session	Short-term relief only	Maniquis-Smigel et al., 2017 [59]; Yildirim et al., 2021; Chen et al., 2023 [63]
	Multiple sessions	Sustained improvements in pain and function	Köroğlu et al., 2019 [60]
Anatomical target	Facet joint/sacroiliac joint	Favorable outcomes	Yildirim et al., 2021 [61]; Raissi et al., 2022 [62]
	Epidural	Rapid but short-term improvement	Maniquis-Smigel et al., 2017 [59], Mohamed et al. [65]
	Multiple targets	Modest to sustained improvement	Köroğlu et al., 2019 [60], Chen et al., 2023 [63]
Guidance modality	Image-guided (ultrasound and/or fluoroscopy)	Consistent and lasting outcomes	Raissi et al., 2022 [62]; Chen et al., 2023 [63]; Mohamed et al., 2024 [65]
	Anatomical land-mark guided	Variable outcomes, potential inaccuracy	Köroğlu et al., 2019 [60]; Yildirim et al., 2021 [61]

Taken together, the findings suggest that optimal outcomes in CLBP are most likely achieved when prolotherapy is applied in multiple sessions, using low-to-intermediate dextrose concentrations, targeting clinically relevant anatomical structures, and using image guidance.

### 4.12. Summary of Findings and Certainty of Evidence (GRADE Assessment)

This systematic review included seven studies assessing the effect of dextrose prolotherapy on CLBP, covering multiple outcomes such as pain intensity, functional disability, quality of life, walking distance, and pain-related daily activity impact. The overall quality of evidence was low to very low, as rated by the GRADE approach (Table 5). Pain intensity was measured using VAS (six studies) and NRS (one study). The NRS-based randomized double-blind trial showed very serious imprecision, resulting in very low certainty. Functional disability was assessed using ODI (three studies: one RCT, two retrospective), with very low certainty due to risk of bias, inconsistency, and imprecision. RMQ was used in a single RCT, also rated very low certainty due to limited sample size and wide confidence intervals. Quality of life (SF-36), assessed in one RCT, showed very low certainty from serious risk of bias and imprecision. Walking distance before pain onset, based on one retrospective study, was downgraded for serious bias and imprecision, with very low certainty. Pain-related daily activity limitation (DPQ) was assessed in one RCT and downgraded for very serious imprecision and suspected publication bias, resulting in very low certainty. Overall, while dextrose prolotherapy may offer benefits for chronic low back pain, current evidence remains very uncertain, highlighting the need for robust, large-scale randomized trials.

### 4.13. Limitations of the Evidence Included in the Review

The evidence on prolotherapy (dextrose prolotherapy) for chronic low back pain presents several important limitations. First, the included studies had small sample sizes, reducing the generalizability of the findings. Moreover, blinding was not consistently assessed or applied across all studies, increasing the risk of bias. Additionally, follow-up periods were often short, limiting the ability to assess long-term efficacy and safety. The lack of standardization regarding the most effective prolotherapy dosage and injection protocols further complicates the interpretation and comparison of results. Notably, the included studies primarily used subjective scales to assess pain and functional outcomes during the follow-up period. Another important limitation concerns the heterogeneity of the underlying causes of chronic low back pain in the included studies. The reviewed trials addressed diverse etiologies, such as disc herniation, lumbar spinal stenosis, facet joint arthropathy, sacroiliac joint dysfunction, or failed back surgery syndrome. These conditions may respond differently to prolotherapy, and grouping them together may limit the detection of effects specific to each condition. As a result, the findings may not be uniformly generalizable across all groups of patients. Furthermore, prolotherapy (dextrose prolotherapy) requires a high level of clinical skill and vigilance to ensure accurate assessment, which may affect the consistency and reliability of outcome measurements across studies and must take into consideration specific etiological subtypes to better identify which patients are most likely to benefit from this intervention.

### 4.14. Limitations of the Review Processes Used

A limitation of this systematic review is the exclusion of non-English studies, which may have led to the omission of relevant data. In addition, the exclusion of other systematic reviews might have resulted in missing important information.

### 4.15. Implications of the Results for Practice, Policy, and Future Research

By employing a reproducible methodology for study selection and data extraction and following PRISMA guidelines, this review ensures transparency. Conducting a comprehensive search across six databases enhances the reliability of the findings and provides a starting point for future research. There is a clear need for high-quality, large-scale randomized controlled trials that apply standardized dosing protocols, consistently target specific anatomical sites [75] and incorporate objective pain measures in addition to subjective pain assessment questionnaires [76]. Furthermore, future studies should investigate the potential benefits of combining prolotherapy (dextrose prolotherapy) with rehabilitation programs. Additional research is essential to better understand the role of this regenerative treatment in managing CLBP.

Considering these findings, it is also important to place dextrose prolotherapy within the wider framework of multidisciplinary chronic pain management. Chronic low back pain is increasingly regarded as a complex condition requiring an integrated approach that may include physical rehabilitation, psychological support, pharmacologic treatment, and interventional procedures. As a minimally invasive and regenerative technique, dextrose prolotherapy may act as a complementary option within multimodal care, especially in patients with persistent pain despite conservative therapies. This perspective aligns with recent recommendations promoting individualized, interdisciplinary strategies in chronic pain management [77].

## 5. Conclusions

Dextrose prolotherapy, as a regenerative treatment, may offer benefits in the management of CLBP, but current evidence remains limited. Most studies have small sample sizes and lack long-term follow-up, reducing the strength and reliability of the findings. Some studies indicate greater benefits following multiple injection sessions, suggesting that treatment frequency could play a role in therapeutic effectiveness. There is no consensus on optimal dosing, injection techniques, or anatomical targets, all of which may influence outcomes. The frequent use of subjective pain scales also highlights the need for more objective evaluation tools. Further research, including larger, high-quality randomized controlled trials and standardized protocols, is necessary to better understand the efficacy and appropriate use of prolotherapy in the context of CLBP.

## Figures and Tables

**Table 1:** Keyword combinations used to search the international databases.

Keyword Combinations	PubMed	Science Direct	Scopus	Springer Nature	Web of Science	Cochrane	Total
Prolotherapy AND low back pain	2	19	59	89	25	0	194
Hypertonic glucose AND low back pain	1	25	5	246	4	0	281
Dextrose prolotherapy AND low back pain	2	7	20	47	11	0	87
D-glucose prolotherapy AND low back pain	3	2	0	13	2	0	20
Total	8	53	84	395	42	0	582

**Table 3:** Risk of bias assessment using PEDro for the included studies.

Authors and Publication Year	Mean Grading											
	1	2	3	4	5	6	7	8	9	10	11	Total *
Liza Maniquis-Smigel et al., 2017 [59]	Yes	Yes	Yes	Yes	Yes	Yes	Yes	Yes	No	Yes	Yes	9/10
Özlem Köroğlu et al., 2019 [60]	Yes	No	No	Yes	No	No	No	Yes	Yes	Yes	Yes	5/10
Timur Yildirim et al., 2021 [61]	Yes	No	No	No	No	No	No	No	Yes	Yes	Yes	3/10
Gholamreza Raissi et al., 2022 [62]	Yes	Yes	No	Yes	Yes	Yes	Yes	Yes	No	Yes	Yes	8/10
Carl PC Chen et al., 2023 [63]	Yes	No	No	Yes	No	No	No	Yes	No	Yes	Yes	4/10
Jose Alberto Pereira Pires et al., 2023 [64]	Yes	Yes	No	Yes	No	No	No	Yes	Yes	Yes	Yes	6/10
Yasmine Ragab Elsayed Mohamed et al., 2024 [65]	Yes	Yes	No	Yes	Yes	No	Yes	Yes	No	Yes	Yes	7/10

* Only criteria 2–11 are scored.

**Table 5:** Summary of findings (GRADE Assessment).

Outcomes	Anticipated Absolute Effects (95% CI)		Relative Effect (95% CI)	№ of Participants (Studies)	Certainty of the Evidence (GRADE)
	Risk with [Comparison]	Risk with [Intervention]			
Pain Evaluation assessed with VAS	The mean pain Evaluation was 0	0 (0 to 0)	-	586 (6 studies)	-^a,b,c,d^
Pain Evaluation assessed with NRS	The mean pain Evaluation was 0	0 (0 to 0)	-	35 (1 RCT)	-^r^
Pain characterization assessed with MPQ	The mean pain characterization was 0	0 (0 to 0)	-	83 (1 RCT)	-^n^
Pain-related impact on daily activities assessed with DPQ	The mean pain-related impact on daily activities was 0	0 (0 to 0)	-	36 (1 RCT)	⨁◯◯◯ * ^e,f,g^
Functional disability assessed with ODI	The mean functional disability was 0	0 (0 to 0)	-	301 (3 studies)	-^h,i,j,k^
Functional disability assessed with RMDQ	The mean functional disability was 0	0 (0 to 0)	-	38 (1 RCT)	-^s^
Quality of life assessed with Sf-36	The mean quality of life was 0	0 (0 to 0)	-	40 (1 study)	-^l,m^
Walking distance assessed with meters before calf pain	The mean walking distance was 0	0 (0 to 0)	-	211 (1 study)	-^o,p,q^

Explanations: a. Study design variability, b. variable results across studies, c. Downgraded due to imprecision: small sample sizes and wide confidence intervals across included studies, d. Publication bias was strongly suspected due to the small number of included studies and the predominance of positive findings or moderate improvement, with a lack of unpublished or negative results, e. Only one study reported DPQ outcomes, limiting the ability to assess inconsistency or precision. Risk of publication bias is strongly suspected due to selective reporting, f. Imprecision was rated very serious due to small sample size and lack of replication, g. Only one positive study available; publication bias cannot be excluded due to lack of confirmatory evidence, h. Half of the studies were retrospective, lacking randomization or blinding, increasing risk of selection and performance bias, i. Results varied in magnitude and timing of ODI/RMDQ improvements across studies, j. Some included studies had small sample sizes, and confidence intervals were wide, k. Positive findings dominate the literature; potential publication bias cannot be excluded, l. Single study with unclear blinding and allocation concealment, m. Single study with small sample size and wide confidence intervals, n. The study had a small sample size and wide confidence intervals, limiting the precision and robustness of the findings, o. The retrospective nature of the study and absence of randomization increase the risk of selection and measurement bias, p. Inconsistency could not be assessed as only one study was included for this outcome, q. Very serious imprecision due to small sample size and absence of confidence interval reporting, r. Small sample size, wide confidence intervals, s. Small sample size and wide confidence intervals. * Very low.

## Abbreviations

CLBP	Chronic low back pain
D5W	Dextrose 5% in Water
DPQ	Dallas Pain Questionnaire
FBSS	Failed Back Surgery Syndrome
GBD	Global Burden of Disease
LBP	Low back pain
LSS	Lumbar Spinal Stenosis
mL	milliliter
MPQ	McGill Pain Questionnaire
NRS	numerical rating scale
NSAIDs	non-steroidal anti-inflammatory drugs
ODI	Oswestry Disability Index
PRISMA	Preferred Reporting Items for Systematic Reviews and Meta-Analyses
PROSPERO	International Prospective Register of Systematic Reviews
RCT	Randomized double-blind clinical trial
RoB 2	Risk of Bias 2
SF-36	36-Item Short Form Survey
SI	sacroiliac
SIJD	Sacroiliac joint dysfunction
TENS	Transcutaneous Electrical Nerve Stimulation
USA	The United States of America
USG	Ultrasound-guided
US	Ultrasound
VAS	Visual Analog Scale
YLDs	years lived with disability
